# Involvement of the *Actinobacillus pleuropneumoniae ompW* Gene in Confrontation of Environmental Pressure

**DOI:** 10.3389/fvets.2022.846322

**Published:** 2022-05-19

**Authors:** Xiabing Chen, Zhiyong Shao, Lijun Wu, Bin He, Wenhai Yang, Jie Chen, Erguang Jin, Qi Huang, Liancheng Lei, Jiajia Xu, Haotian Li, Hui Zhang, Yun Wan, Wu Liu, Rui Zhou

**Affiliations:** ^1^Institute of Animal Husbandry and Veterinary Science, Wuhan Academy of Agricultural Sciences, Wuhan, China; ^2^State Key Laboratory of Agricultural Microbiology, College of Animal Science and Veterinary Medicine, Huazhong Agricultural University, Wuhan, China; ^3^Cooperative Innovation Center for Sustainable Pig Production, College of Animal Science and Veterinary Medicine, Huazhong Agricultural University, Wuhan, China; ^4^College of Veterinary Medicine and College of Animal Science, Jilin University, Changchun, China; ^5^Wuhan Animal Disease Control Center, Wuhan, China

**Keywords:** *A. pleuropneumoniae*, *ompW*, susceptibility, phenotype, infections

## Abstract

Actinobacillus pleuropneumoniae causes porcine pleuropneumonia. The function of the outer membrane protein W gene (*ompW*) of *A. pleuropneumoniae* has not been evaluated. Thus a deletion mutant of *ompW*, Δ*ompW*, was constructed to explore the effect of *ompW* gene deletion on bacterial growth, biofilm formation, bacterial morphology, oxidative tolerance, susceptibility to antibiotics, and the expression of ribosome synthesis and ABC transporter related genes. Results showed that the *ompW* gene deletion did not affect biofilm formation and the growth of *A. pleuropneumoniae* but did affect bacterial morphology during steady growth, oxidative tolerance, and bacterial susceptibility to polymyxin B, kanamycin, and penicillin. The *ompW* gene deletion also affected the expression of ribosome synthesis and ABC transporter related genes. These results suggested that *ompW* may regulate the biological phenotype of *A. pleuropneumoniae*.

## Introduction

*Actinobacillus pleuropneumoniae* causes porcine pleuropneumonia and is one of the most important bacterial respiratory pathogens of pigs (1). In large-scale pig breeding farms, economic loss due to *A. pleuropneumoniae* is a serious concern. Based on the growth requirement for nicotinamide adenine dinucleotide (NAD), *A. pleuropneumoniae* are identified as biotype I or biotype II (2). Biotype I strictly requires NAD for growth, while biotype II does not require NAD as a growth supplement (3). Based on the nature of the bacterial capsule and lipopolysaccharide, *A. pleuropneumoniae* is classified into 19 serotypes, with serotypes 1, 5, 9, 10, and 11 highly virulent (4, 5). In China, serotypes 1, 3, and 7 are the dominant serovars (6). Immunological protection does not exist across serotypes (7), hence *A. pleuropneumoniae* infections are difficult clinical problems.

Recently, important virulence related factors have been identified that are involved in the pathogenesis of *A. pleuropneumoniae*. Repeats-in-toxin (RTX) and ApxIIIA toxin have been shown to target host cell β2 integrins killing many types of leukocytes and phagocytic cells (8). Deletion of PEP-carboxylase (PEPC) and PEP-carboxykinase (PEPCK) attenuate *A. pleuropneumoniae* virulence in a pig infection model (9). Disruption of TolC1 significantly reduces *A. pleuropneumoniae* virulence in a murine intraperitoneal injection model (10). Inactivation of flp1 and tadD genes affected *A. pleuropneumoniae* biofilm formation, cell adhesion, and resistance to phagocytosis (11). The *hfq* gene of *A. pleuropneumoniae* is helpful to regulate the adhesion to biological and abiotic surfaces, resistance to various stress conditions, and virulence (12). Deletion of outer membrane lipoprotein, Lip40, significantly attenuates adherence to St. Jude porcine lung cells and colonization of mouse lung tissue, indicating that Lip40 participates in the virulence of *A. pleuropneumoniae* (13). CpxA/CpxR affect *A. pleuropneumoniae* biofilm formation with mutation of *cpxA/cpxR* reducing mortality and bacterial load in a murine experimental lung infection model (14). Although these virulence factors have been intensively investigated, the mechanistic basis for *A. pleuropneumoniae* pathogenicity remains unclear.

Outer membrane proteins (OMPs) of bacteria play core roles in bacterial pathogenesis (15). The *ompW* gene is found in many bacteria, such as *Escherichia coli* (16), *Aeromonas hydrophila* (17), and *Vibrio harveyi* (18). *E. coli OmpW* knockout contributes to oxidative stress resistance (19). Response to the *OmpW* protein provides immunity to *Aeromonas veronii* challenge, with an immunization strategy a potential means by which to control *Aeromonas veronii* infection (20). *OmpW* regulates biofilm formation of *Cronobacter sakazakii* during NaCl stress (21). Deletion of *ompW* attenuates *Vibrio cholerae* growth in hypersaline culture conditions (22). *Caulobacter crescentus ompW* is an outer membrane cation channel (23). It serves as a multidrug resistance transporter involved in the efflux of ethidium multidrug resistance protein E (EmrE) substrates across the outer membrane (24). The function of *A. pleuropneumoniae ompW* has not been identified.

The purpose of this study was to investigate the role of *A. pleuropneumoniae ompW* in bacterial pathogenicity. To do so we constructed a deletion mutant of *ompW*, strain Δ*ompW*, and a complemented strain, CΔ*ompW*. Deletion of the *ompW* gene did not affect the growth and biofilm formation of *A. pleuropneumoniae*, but did influence steady state growth bacterial morphology, as well as bacterial susceptibility to polymyxin B, kanamycin, and penicillin. In addition, *ompW* gene inactivation modified expression of ribosome synthesis related genes and ABC transporter genes. These results provide a better understanding of *A. pleuropneumoniae ompW* gene function during infection.

## Materials and Methods

### Bacterial Strains and Growth Conditions

The *A. pleuropneumoniae* 4074 strain, serovar 1, is highly virulent and cultured on tryptic soy agar (TSA; Difco Laboratories, USA) or in tryptic soy broth (TSB, Difco Laboratories, USA) supplemented with 10% fetal bovine serum (FBS) (Gibco, USA) and 10 μg/mL NAD (Sigma-Aldrich, USA). The Δ*ompW* and CΔ*ompW* strains were constructed in this study, and the culture conditions were the same as the *A. pleuropneumoniae* 4074 strain. The pEMOC2 plasmid was from the laboratory of Prof. gerald-f. Gerlach and the pJFF224-XN plasmid was stored in our laboratory. The pEMΔ*ompW* and pJFF-*ompW* plasmid were constructed in this study. The *E. coli* β2155 strain was cultured on Luria-Bertani (LB, Oxoid Ltd. UK) agar or in broth. The *A. pleuropneumoniae* and *E. coli* strains were cultured at 37°C. If necessary, 2 μg/mL or 50 μg/mL of chloramphenicol (Sigma-Aldrich, USA) was added to the culture medium.

### Construction of the *A. pleuropneumoniae ompW* Gene Deletion Mutant, *ΔompW*, and the *OmpW* Gene Deletion Complemented Mutant, C*ΔompW*

The method for construction of Δ*ompW* has been reported previously (25). Briefly, a 648 bp internal deletion of the *ompW* gene was amplified with primers *ompW*UF/*ompW*UR and *ompW*DF/*ompW*DR utilizing single-overlap extension polymerase chain reaction (SOE PCR). Then a 2073 bp PCR product (pE*ompW*UF/pE*ompW*UR) was cloned into the suicide plasmid vector pEMOC2 to obtain the plasmid pEMΔ*ompW* (26), which included the 648 bp deletion fragment in frame. The pEMΔ*ompW* plasmid in E. coli β2155 strain was transformed into the wild-type strain of *A. pleuropneumoniae* with a single-step transconjugation system (27, 28). The Δ*ompW* mutant was obtained by two homologous recombination steps and was identified with *ompW*MF/*ompW*MR primers.

The *ompW* gene was amplified with *ompW*F/*ompW*R primers for construction of CΔ*ompW*. The *ompW* gene was then connected to the shuttle vector pJFF224-XN and was electrically transferred into the Δ*ompW* mutant to construct the corresponding complemented strain. Voltage was 2.5 KV, capacitance 25 μFD, and pulse resistance 800 Ω using an electroporation apparatus (Bio-Rad, USA). The complemented strain was screened with 2 μg/mL chloramphenicol and was identified with PJ*ompW*R/ PJ*ompW*F primers. The bacterial strains, plasmids used in this study are listed in Table 1 and the primers used in this study are listed in Table 2.

**Table 1:** Bacterial strains and plasmids used in this study.

**Strains/plasmids**	**Relevant characteristics**	**Source of references**
*A. pleuropneumoniae* 4074	high toxic strain	Laboratory stock
*ΔompW*	*ompW* gene knockout mutant	This work
C*ΔompW*	Complemented strain of *ΔompW*	This work
β2155	*thrB1004 pro thi strA hsdS lac*ZΔM15 (F' *lacZ*ΔM15 *lacl*^q^ *traD*36*proA*^+^ *proB*^+^)*Δdap*:: *erm* (Erm^r^))*recA:*: *RPA-2-tet*(Tc^r^)::Mu-km (Km^r^) λ*pir*	From Prof. Gerald-F. Gerlach
pEMOC2	Conjugative vector based on pBluescript SK with mobRP4, polycloning site, *Cm^*r*^*, and transcriptional fusion of the *omlA* promoter with the *ompW* gene	From Prof. Gerald-F. Gerlach
pEM*ΔompW*	Conjugative vector pEMOC2 with a 648bp deletion in the *ompW* gene which have a 1065 bp upstream fragment and 1008 bp downstream fragment	This work
pJFF224-XN	*E. coli*-APP shuttle vector: RSF1010 replicon; mob oriV, Cm^r^	Laboratory stock
pJFF-*ompW*	pJFF224-XN carrying the intact *ompW*	This work

**Table 2:** Primers used in this study.

**Gene**	**Primer**	**Sequence**
kdsB	kdsB-F	5'-CAATCCGAATGCCGTCAAA-3'
	kdsB-R	5'-CGGCGCACGAGAGAAATAG-3'
rpmA	rpmA-F	5'-GGTTCAACTCGTAACGGTCG-3'
	rpmA-R	5'-TTTTCTCGCCTTTCACTTCAAA-3'
rpmB	rpmB-F	5'-AGAGTTTGCCAAGTAACCGG-3'
	rpmB-R	5'-ACGCATACCTTTCGCTGTTA-3'
xylG	xylG-F	5'-TCGCTTTATCCGCATGAACC-3'
	xylG-R	5'-AACCGAAGATACATTGCGCC-3'
modA	modA-F	5'- CCGGCAGCTTTAAATTTCGC-3'
	modA-R	5'- GTTTGTCGAACGTGGGGAAT-3'
rplT	rplT-F	5'-AGAGCACGCCATAAGAAAGT-3'
	rplT-R	5'-AGCCGTTGATGAATTTGCTGT-3'
rpsA	rpsA-F	5'-GCTTCTTCACCTGTCGCATT-3'
	rpsA-R	5'- GAACACTTCGCAGCAACTCA-3'
malK_1	malK_1-F	5'-CGCTTAATAATGCCCTGCCA-3'
	malK_1-R	5'-TAATCTCGCACCGGAAAAGC-3'
phnS_2	phnS_2-F	5'-TCGTTGATTTCGCGCTTTCT-3'
	phnS_2-R	5'-TTACGTTCGTCACTTGCACC-3'
rpsC	rpsC-F	5'-AACTATTCACACAGCGCGTC-3'
	rpsC-R	5'-CGCGACGTTCAAGTTGAGAA-3'
rplU	rplU-F	5'-AACTTGAAATCGCAACTGGTG-3'
	rplU-R	5'-ACCACTGACGATGACCTTGT-3'
fhuB	fhuB-F	5'-GGATTTTACTGTCGGTCGCC-3'
	fhuB-R	5'-AATCCCTGCCAACCAAAACC-3'
PROKKA_00896	896-F	5'-TTACTGCCGAAACTGACCGA-3'
	896-R	5'-ACGTACCTTCCGCCGAATAT-3'
rplC	rplC-F	5'-TCCGTACCCAAGATGCTACC-3'
	rplC-R	5'-CGCTCAGCATCTACACGAAC-3'
	*ompW*UF	5'-TTCTTCTTTGGCTAATTCGTTAAG-3'
	*ompW*UR	5'-TCATAACAGTTTCAAAATACTTTGACCTCTTAATATTTTCTTA-3'
	*ompW*DF	5'-GAAAATATTAAGAGGTCAAAGTATTTTGAAACTGTTATGAGAA-3'
	*ompW*DR	5'-TATTATTAGCCGGTCATACCGA-3'
	pE*ompW*UF	5'-ATTTGCGGCCGCTTCTTCTTTGGCTAATTCGTTAAG-3'(Not I)
	pE*ompW*DR	5'-ACGCGTCGACTATTATTAGCCGGTCATACCGA-3'(Sal I)
	*ompW*MF	5'-GGGCTTCTTGCAATTTGG-3'
	*ompW*MR	5'-CGCTCCCTTTCTCATAAC-3'
*ompW*	*ompW*F	5'-GTCGCGGATCCATGAAAAAAGCAGTATTAGCG-3'(BamH I)
	*ompW*R	5'-CTAGTCTAGATTAGAATTTGTAGCTAATACCTG−3'(Xba I)
	PJ*ompW*F	5'-AAGACCCAGAGTATTGCGA-3'
	PJ*ompW*R	5'-CATTGCCTGGTTGCTTCAT-3'

### The Effect of the *OmpW* Gene Deletion on *A. pleuropneumoniae* Growth Kinetics

The effect of the *ompW* gene deletion on *A. pleuropneumoniae* growth was assessed as described previously (29). Briefly, the *A. pleuropneumoniae*, the Δ*ompW*, and CΔ*ompW* strains were cultured in TSB supplemented with 10% FBS and 10 μg/mL NAD at 37°C overnight. The bacterial were then transferred to fresh TSB supplemented with 10% FBS and 10 μg/mL NAD at a 1:100 (V/V) dilution for logarithmic growth. The initial OD600 value was adjusted to 0.2 and the OD600 value of the bacterial suspension determined 1 h to 8 h after culture initiation for kinetic analysis.

### Determination of the Effect of the *OmpW* Gene Deletion on *A. pleuropneumoniae* Biofilm Formation

The *A. pleuropneumoniae* and the Δ*ompW* biofilm formation were as described previously with minor modifications (30). Briefly, an overnight bacterial culture was transferred to fresh TSB supplemented with 10% FBS and 10 μg/mL NAD at a 1:100 (V/V) dilution. Then a 100 μL bacterial suspension was added to the wells of a 96-well microplate and incubated for 12 h, 24 h, 36 h, and 48 h at 37°C. The bacterial culture liquid was removed, each well washed with PBS, and 100 μL of Crystal Violet dye solution (0.1%) added for 30 min. The wells were washed with PBS to remove unbound crystal violet dye and dried for 5 h at 37°C. Then 100 μL of a glacial acetic acid solution (33%, v/v) was added to each well and the microplate was shaken for 1 h. The absorbance at 590 nm was measured.

### Determination of the Effect of the *OmpW* Gene Deletion on the Bacterial Morphology of *A. pleuropneumoniae* by Scanning Electron Microscopy (SEM) and Transmission Electron Microscopy (TEM)

The bacterial morphologies of the *A. pleuropneumoniae* and the Δ*ompW* strains were examined by SEM and TEM as described previously with minor modifications (31, 32). Briefly, the strains were cultured to logarithmic and steady state phases of growth. The bacteria were collected, washed three times with PBS, and fixed with 4% glutaraldehyde. Specimens were dehydrated, dried, and sprayed with gold. Bacterial morphologies were observed with a HITACHI SU8010 scanning electron microscope (Hitachi, Japan). For TEM, the strains were fixed with 0.1 M cacodylate buffer containing 5% glutaraldehyde and 0.15% ruthenium red at 37°C for 4 h and then treated with 1 mg/mL polycationic ferritin. Thin sections were prepared with a Tecnai G^2^ 20 TWIN transmission electron microscope (FEI, Portland, OR, USA).

### Oxidative Tolerance Test

Stress resistance tests were conducted using the *A. pleuropneumoniae*, the Δ*ompW*, and CΔ*ompW* strains. These strains were grown in TSA supplemented with 10% FBS and 10 μg/mL NAD at 37°C overnight. The bacterial were then transferred to fresh TSB supplemented with 10% FBS and 10 μg/mL NAD at a 1:100 (V/V) dilution for logarithmic growth. At an OD600 value of approximately 0.6, cells from 1 ml of broth cultures were centrifuged at 5000 g for 5 min. For the oxidative tolerance test, the cells were resuspended in 1 ml of TSB supplemented with 10% FBS, 10 μg/mL NAD and 1 mM hydrogen peroxide for 10 min or 20 min, respectively. The control samples of each strain were resuspended in 1 ml of TSB supplemented with 10% FBS and 10 μg/mL NAD without any treatment. Then, the cultures from oxidative stress resistance test were serially diluted in TSB, and spread on TSB plates supplemented with 10% FBS and 10 μg/mL NAD for CFU counting. Oxidative stress resistance was calculated as ((oxidative stressed sample CFU mL^−1^)/(control sample CFU mL^−1^)) × 100. The experiments were carried out in triplicate (20).

### Determination of the Effect of the *OmpW* Gene Deletion on *A. pleuropneumoniae* Antibiotic Susceptibility

The minimal inhibitory concentration (MIC) of the *A. pleuropneumoniae*, Δ*ompW*, and CΔ*ompW* strains for 19 antibiotics (rifampicin, Ofloxacin, polymyxin B, vancomycin, acriflavine, cefalexin, lincomycin, gentamicin, kanamycin, neomycin, penicillin, amoxicillin, tetracycline, streptomycin, vibramycin, enrofloxacin, florfenicol, sulfamethoxazole, and ceftiofur sodium; Yuanye biology Co., Ltd, China) was determined as described previously with minor modifications (33). Briefly, 50 μL of a TSB culture supplemented with 10% FBS and 10 μg/mL NAD of each strain were added to 96-well plates. The appropriate concentration of antibiotics was diluted 12 times continuously, and then 50 μL of diluted antibiotics were added to the 96 well plate to make the final concentration of 0.125 to 256 μg/mL or 0.015625 to 32 μg/mL. Bacterial concentration was adjusted to a 0.5 Macbeth turbidity using 5 mL of sterile normal saline. Then 100 μL the bacterial suspension was added to 10 mL TSB supplemented with 10% FBS and 10 μg/mL NAD for measurement. The positive control (with bacteria and without drugs) and the negative control (without bacteria and drugs) were assessed by adding 50 μL of the tested bacterial solution to each antibiotic well. The tested bacterial solution was cultured at 37°C for 24 h. The highest concentration without bacterial growth was observed as the MIC of the antibiotic and recorded.

### RT-PCR Determination of the Effect of the *OmpW* Gene Deletion on Genes Involved in the Ribosome Synthesis Pathway and the ABC Transporter Pathway

To explore the effect of the *ompW* gene deletion on the expression levels of chose genes, total RNA of bacteria was extracted by TRIzol reagent (Invitrogen, USA) (34). The strains cultured in TSB supplemented with 10% fetal bovine serumand 10 μg/mL NAD. The RNA was reverse-transcribed to cDNA with a PrimeScript^TM^ II 1st Strand cDNA kit (Takara, Dalian, China). Amplification of cDNA was carried out using the SYBR Premix Ex Taq kit (Takara, Dalian, China). The *rpmA, rpmB, xylG, modA, rplT, rpsA, malK_1, phnS_2, rpsC, rplU, fhuB, prokka_00896, rplC* genes were analyzed and the *kdsB* gene encoding 3-deoxy-manno-octulosonate cytidylyltransferase was used as the internal control (35). The primers used for RT-PCR are found in Table 2.

### Statistical Analysis

Experimental data are presented as means ± SD. The difference between two groups was analyzed by the two-tailed Student's *t*-test. *P* values of < 0.05 were considered significant, ^*^p < 0.05 and ^**^p < 0.01.

## Results

### Construction of the *OmpW* Gene Deletion Mutant, *ΔompW, and the* Complemented Strain of the *OmpW* Gene Deletion Mutant, C*ΔompW*

**Figure 1:**
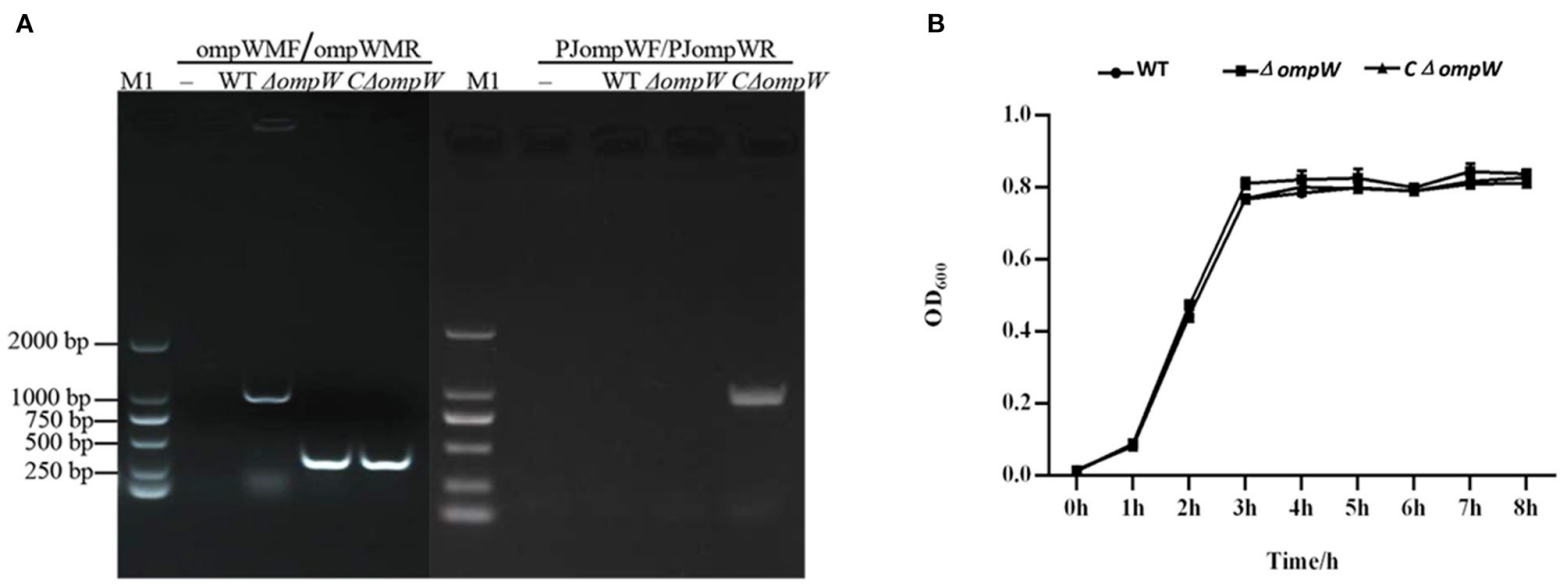
**(A)** Confirmation of the Δ*ompW* mutant and CΔ*ompW* by PCR using primer pairs. M1: 2000 bp marker; WT: wild type strain of *A. pleuropneumoniae*. **(B)** Growth curves for the *A. pleuropneumoniae* strain, the Δ*ompW* mutant strain, and the CΔ*ompW* strain, cultured to logarithmic growth phase. The OD_600_ values of bacterial solutions are presented.

The *ompW* gene deletion mutant, Δ*ompW*, was constructed by homologous recombination and verified by PCR (Figure 1A). The CΔ*ompW* strain was confirmed with PJ*ompW*F/PJ*ompW*R (Figure 1A).

### The *OmpW* Gene Deletion Did Not Affect the Growth of *A. pleuropneumoniae in vitro*

The *in vitro* effect of the *ompW* gene deletion on 8 h growth was assessed for the *A. pleuropneumoniae* strain, the Δ*ompW* mutant strain, and the CΔ*ompW* strain. Results showed the growth kinetics of the *A. pleuropneumoniae*, the Δ*ompW* mutant strain, and the *C*Δ*ompW* strain to not differ significantly for this incubation period (Figure 1B).

### Deletion of the *OmpW* Gene Did Not Affected Biofilm Formation by *A. pleuropneumoniae in vitro*

**Figure 2:**
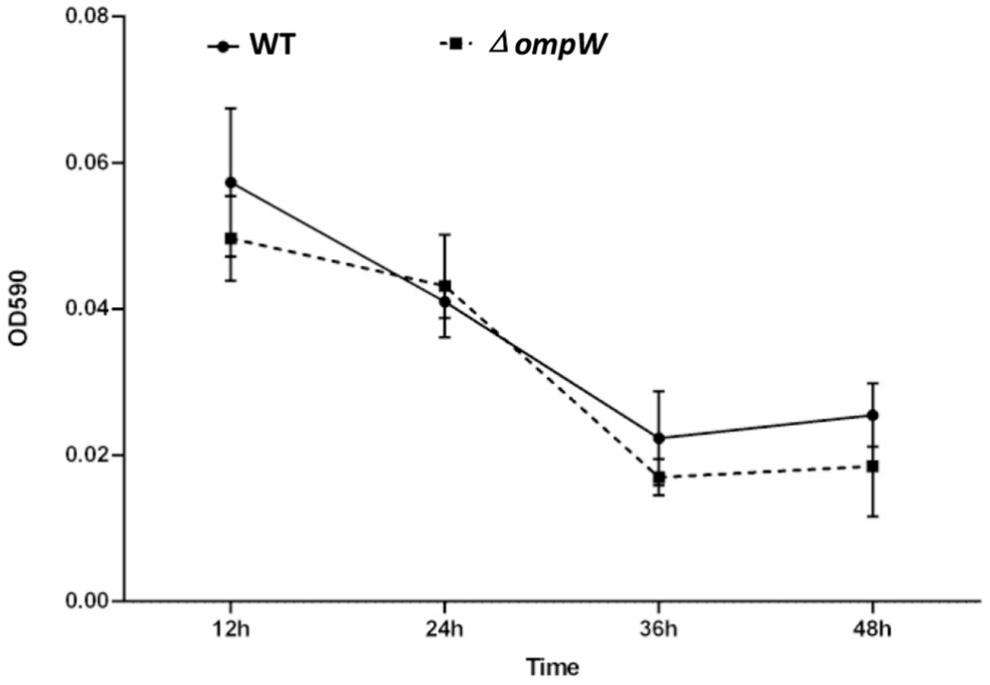
Biofilms formation by the *A. pleuropneumoniae* strain and the Δ*ompW* mutant strain. Liquid was removed after static incubation at 37°C followed by the addition of a crystal violet dye solution. The biofilms were dissolved by adding a glacial acetic acid solution and the OD590 absorbance of each well determined. The experiment has at least three biological repetitions.

We explored the effect of *ompW* gene deletion on biofilm formation of *A. pleuropneumoniae* and Δ*ompW* using a quantitative assay. The results showed that deletion of *ompW* gene reduced the biofilm formation ability of *A. pleuropneumoniae* at 12 h to 48 h, but the difference is not significant (Figure 2).

### The Effect of the *OmpW* Gene Deletion on the Bacterial Morphology of *A. pleuropneumoniae* During Logarithmic and Steady State Growth

**Figure 3:**
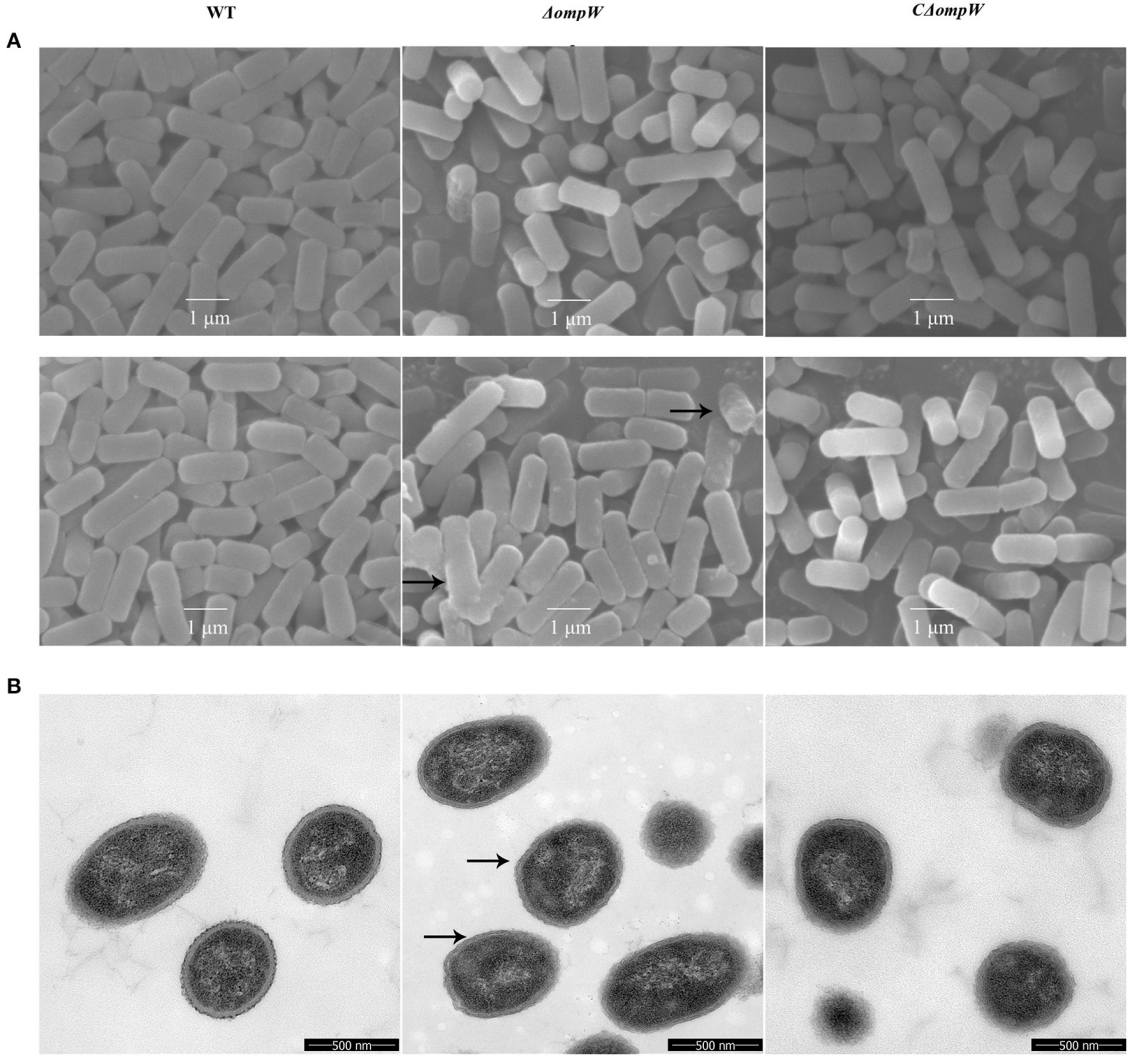
**(A)** SEM and **(B)** TEM morphology of the *A. pleuropneumoniae*, Δ*ompW* mutant strain, and CΔ*ompW* strain during logarithmic and steady state growth.

SEM and TEM morphologies were assessed for the *A. pleuropneumoniae* strain, the Δ*ompW* mutant strain, and the CΔ*ompW* strain during logarithmic and steady state growth. There were no significant morphological differences for any of the strains during the logarithmic growth phase as judged by SEM (Figure 3A). However, for steady growth, the cell surface of the Δ*ompW* mutant became rough, while the surfaces of the *A. pleuropneumoniae* strain and the CΔ*ompW* strain were smooth by SEM (Figure 3A), which indicated absence of the *ompW* gene resulted in a rough surface for *A. pleuropneumoniae* during steady growth. These surface morphologies were confirmed by TEM (Figure 3B).

### *OmpW* Is Required for *A. Pleuropneumoniae* Oxidative Stress Tolerance

**Figure 4:**
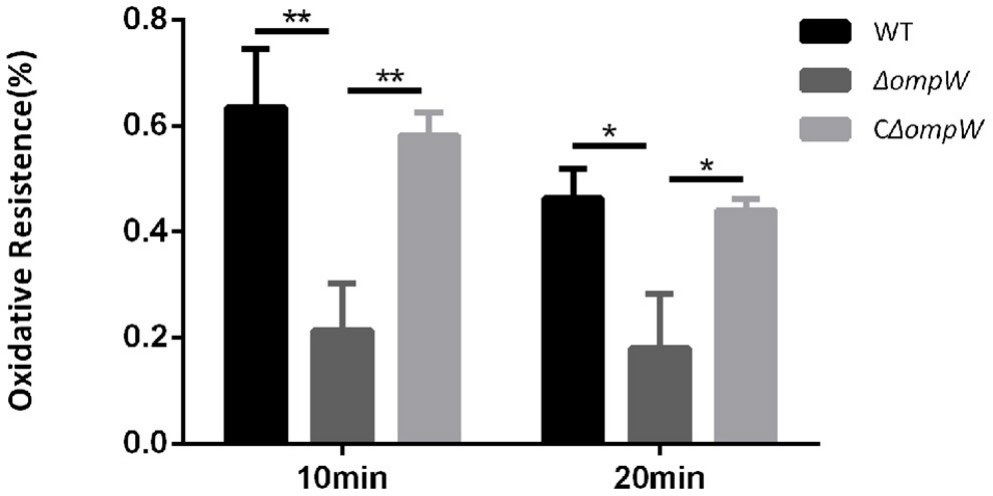
Impaired oxidative stress tolerance of the *A. pleuropneumoniae* strain, the Δ*ompW* mutant strain, and the CΔ*ompW* strain. Overnight cultures were inoculated into fresh TSB supplemented with 10% FBS and 10 μg/mL NAD and grown to an OD_600_ value of approximately 0.6. Cells were then treated with 1 mM hydrogen peroxide for 10 min or 20 min, respectively. The experiment has at least three biological repetitions. Results are expressed as means ± SD of three independent experiments. ‘*' indicates significance at *p* < 0.05 and ‘**' indicates significance at *p* < 0.01.

The *A. pleuropneumoniae*, Δ*ompW*, and CΔ*ompW* strains were exposed to oxidative stress conditions. When the strains were treated with 1 mM hydrogen peroxide for 10 min, the Δ*ompW* strain survival rate was 0.21%, which was much lower than that of the *A. pleuropneumoniae* strain (0.64%) and the CΔ*ompW* strain (0.58%; Figure 4). When the strains were treated with 1 mM hydrogen peroxide for 20 min, the Δ*ompW* strain survival rate was 0.18%, which was lower than that of the *A. pleuropneumoniae* strain (0.46%) and the CΔ*ompW* strain (0.44%). These results suggest that OmpW has a role in the tolerance of *A. pleuropneumoniae* to oxidative stress.

### Deletion of *OmpW* Affected Susceptibility for Polymyxin B, Kanamycin, and Penicillin

The *A. pleuropneumoniae*, Δ*ompW*, and CΔ*ompW* strains were assessed for bacterial susceptibility to; rifampicin, ofloxacin, polymyxin B, vancomycin, acriflavine, cefalexin, lincomycin, gentamicin, kanamycin, neomycin, penicillin, amoxicillin, tetracycline, streptomycin, vibramycin, enrofloxacin, florfenicol, sulfamethoxazole, and ceftiofur sodium. As judged by MIC, deletion of the Δ*ompW* gene did not affect bacterial susceptibility to most assessed antibiotics (Table 3). However the deletion resulted in two times sensitive to polymyxin B, kanamycin, and penicillin (Table 3).

**Table 3:** Determination of MICs for the three bacterial strains.

**Antibiotic (μg/mL)**	**APP**	***ΔompW***	**C*ΔompW***
Rifampicin	0.5	0.5	0.5
Ofloxacin	0.03125	0.03125	0.03125
Polymyxin B	8	4	4
Vancomycin	128	128	128
Acriflavine	2	2	2
Cefalexin	2	2	2
Lincomycin	16	16	16
Gentamicin	32	32	32
Kanamycin	64	32	64
Neomycin	128	128	128
Penicillin	0.5	0.25	0.5
Amoxicillin	1	1	1
Tetracycline	0.5	0.5	0.5
Streptomycin	128	128	128
Vibramycin	0.5	0.5	0.5
Enrofloxacin	0.015625	0.015625	0.015625
Florfenicol	1	1	1
Sulfamethoxazole	256	256	256
Ceftiofur sodium	0.03125	0.03125	0.03125

### Effect of the *OmpW* Gene Deletion on Ribosome Synthesis Related and ABC Transporter Gene Expression

**Figure 5:**
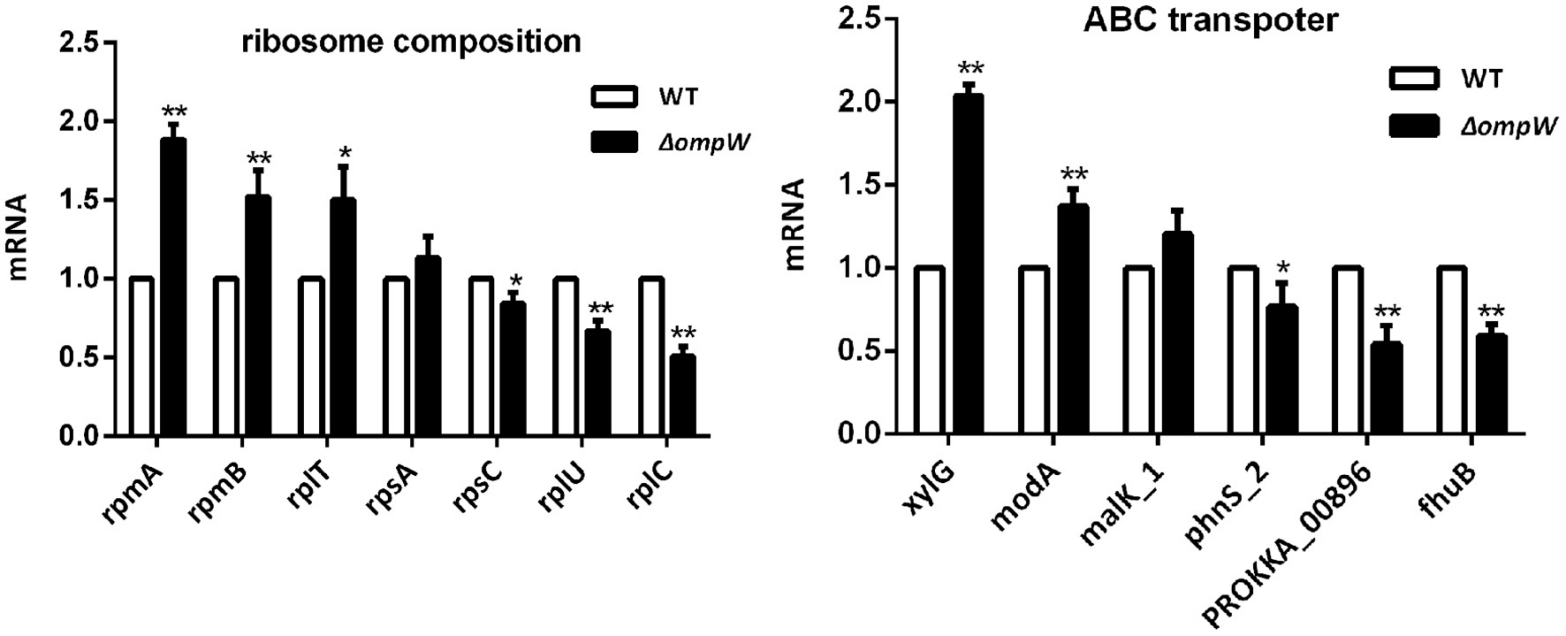
RT-PCR determination of the expression levels of ribosome synthesis related genes and ABC transporter genes following *ompW* gene deletion. Total RNA was extracted from bacterial strains and reverse-transcribed to cDNA. Amplification of cDNA was carried out using SYBR Premix Ex Taq kit. The experiment has at least three biological repetitions. Results are expressed as means ± SD of three independent experiments. ‘*' indicates significance at *p* < 0.05 and ‘**' indicates significance at *p* < 0.01.

The effect of the *A. pleuropneumoniae* and Δ*ompW* strains on ribosome synthesis related genes and ABC transporter genes at the mRNA level was determined by RT-PCR. Three genes, *rpmA, rpmB*, and *rplT* were significantly upregulated and three genes, *rpsC, rplU*, and *rplC* were significantly downregulated within the ribosome synthesis pathway (Figure 5). For the ABC transporter pathway, deletion of the *ompW* gene upregulated *xylG*, and *modA*, while *phnS_2, PROKKA_00896*, and *fhuB* were downregulated (Figure 5).

## Discussion

*A. pleuropneumoniae* is considered one of the most important bacterial respiratory pathogens of pigs with epidemic disease resulting in serious economic loss (36). There are many studies on the infection mechanism and the resistance to environmental pressure of *A. pleuropneumoniae*, but there are still many unknowns to be researched. In this study, we explored the role of the *A. pleuropneumoniae ompW* gene in biofilm formation, resistance to environmental pressure and gene regulation, with the concept that this exploration may provide a novel new strategy for the control of *A. pleuropneumoniae* infections.

Biofilms are a complex community of microorganisms thought to be a sessile mode of life that permits attachment and growth of microorganisms on surfaces (37). Microorganisms comprising biofilms are highly resistant to antibiotics and are capable of prolonged persistence within a host (38), and are involved in virulence and pathogenicity (39). Device-related and chronic infections are often associated with biofilm formation (40). *A. pleuropneumoniae* can produce biofilm that confers bacterial resistance to antibiotics and increased pathogenicity (41–43). Interestingly, the transcription of *ompW* gene and the biofilm formation of *A. pleuropneumoniae* were inhibited by zinc (44). The transcription of *ompW* gene of *A. pleuropneumoniae* is down regulated under iron restriction (45). Studies have shown that *ompW* gene is involved in the protection of bacteria against various forms of environmental pressure. The OmpW was related to salt stress and that *ompW* gene transcription and expression were up-regulated in cultures containing high NaCl concentrations. Deletion of *ompW* gene did not affect the cell morphology of *V. cholerae* (22). Furthermore, iron regulates OmpW by binding to Fur and SoxS, a transcriptional factor involved in oxidative stress, was found to negatively regulate OmpW of *E. coli* (19). In this study, the *ompW* gene was demonstrated to protect *A. pleuropneumoniae* from oxidative stress and affect the cell morphology during steady growth. Whether the *ompW* gene is involved in *A. pleuropneumoniae* virulence requires further study.

Ribosomes are essential to cellular protein production (46). Impairment or abnormalities in the ribosome biogenesis pathways have been related to disease (47). ATP-binding cassette (ABC) transporters use energy derived from ATP hydrolysis for molecular transport across membranes (48) and are involved in many physiological and pathological processes (49). In this study, the ribosome synthesis and ABC transporter pathways were found to have significant involvement. The genes *rpmA, rpmB*, and *rplT* were significantly upregulated and *rpsC, rplU*, and *rplC* were significantly downregulated for the ribosome synthesis pathway. The genes *xylG*, and *modA* were upregulated and *phnS_2, PROKKA_00896, fhuB* were downregulated for the ABC transporter pathway. These genes were chose by our previous RNA-seq results (not mentioned in this study). Research have been shown that these genes have important roles involved in the pathogenicity of *A. pleuropneumoniae*. Thus, we speculate that deletion of the *ompW* gene affected protein synthesis and ion transport, which may affect the toxicity and drug sensitivity of *A. pleuropneumoniae* to a certain extent. *ompW* gene deletion did affect bacterial susceptibility to polymyxin B, kanamycin, and penicillin. The effect on bacterial susceptibility to polymyxin B, kanamycin, and penicillin may also be due to the change of cell membrane permeability. However, the mechanistic basis for these observations requires further investigation.

In summary, data suggested that *ompW* gene deletion did not affect the growth of *A. pleuropneumoniae*, but did affect; morphology during steady growth, oxidative tolerance, and bacterial susceptibility to polymyxin B, kanamycin, and penicillin. The *ompW* gene deletion also altered the expression of ribosome synthesis and ABC transporter related genes. Thus, the *ompW* gene may regulate the biological phenotype of *A. pleuropneumoniae* during *A. pleuropneumoniae* infections.

## Data Availability Statement

The authors acknowledge that the data presented in this study must be deposited and made publicly available in an acceptable repository, prior to publication. Frontiers cannot accept a manuscript that does not adhere to our open data policies.

## Author Contributions

XC, WL, and RZ: designed the experiments. XC, ZS, and LW: performed the experiments. XC, BH, WY, JC, EJ, QH, LL, JX, HL, HZ, YW, WL, and RZ: analyzed the data. XC: wrote the manuscript. All authors contributed to the article and approved the submitted version.

## Conflict of Interest

The authors declare that the research was conducted in the absence of any commercial or financial relationships that could be construed as a potential conflict of interest.

## Publisher's Note

All claims expressed in this article are solely those of the authors and do not necessarily represent those of their affiliated organizations, or those of the publisher, the editors and the reviewers. Any product that may be evaluated in this article, or claim that may be made by its manufacturer, is not guaranteed or endorsed by the publisher.
